# Author Correction: Label-free human skin imaging with enhanced molecular contrast via time-resolved fluorescence and advanced phasor analysis

**DOI:** 10.1038/s42003-026-10469-5

**Published:** 2026-06-11

**Authors:** Suman Ranjit, Belen Torrado, Alexander Vallmitjana, Amanda Fedyk Durkin, Alexander Dvornikov, Anand Ganesan, Kristen M. Kelly, Mihaela Balu

**Affiliations:** 1 Beckman Laser Institute and Medical Clinic, University of California Irvine, Irvine, CA USA; 2 Department of Oncology, Georgetown University Medical Center, Washington, DC USA; 3 Department of Dermatology, University of California Irvine, Irvine, CA USA

**Keywords:** Fluorescence imaging, Diagnostic markers, Biological fluorescence

Correction to: *Communications Biology* **9**:149 (2025); 10.1038/s42003-025-09427-4, published online 30 December 2025

In the version of this article initially published, the statement of competing interests was incomplete and has now been amended to state that “M.B. is a coauthor of a patent owned by the University of California, Irvine (UCI) related to the development of clinical multiphoton microscopy technology. Additionally, M.B. is a cofounder of Infraderm, LLC, a startup spin-off from UCI focused on commercializing clinical multiphoton microscopy imaging platforms that may benefit from the use of advanced analysis tools. The Institutional Review Board and Conflict of Interest Office of UCI have reviewed patent disclosures and found no concerns. The other authors declare no competing interests.” The error has been corrected in the HTML and PDF versions of the article.

