# Supplementary material for: Label-free human skin imaging with enhanced molecular contrast via time-resolved fluorescence and advanced phasor analysis
Source: Commun Biol. 2025 Dec 30;9:149. doi: 10.1038/s42003-025-09427-4 (PMC12868790; doi:10.1038/s42003-025-09427-4)
Supplement: Supplementary file 2 — Supplementary Information [file 42003_2025_9427_MOESM2_ESM.pdf]

Supplemental figures

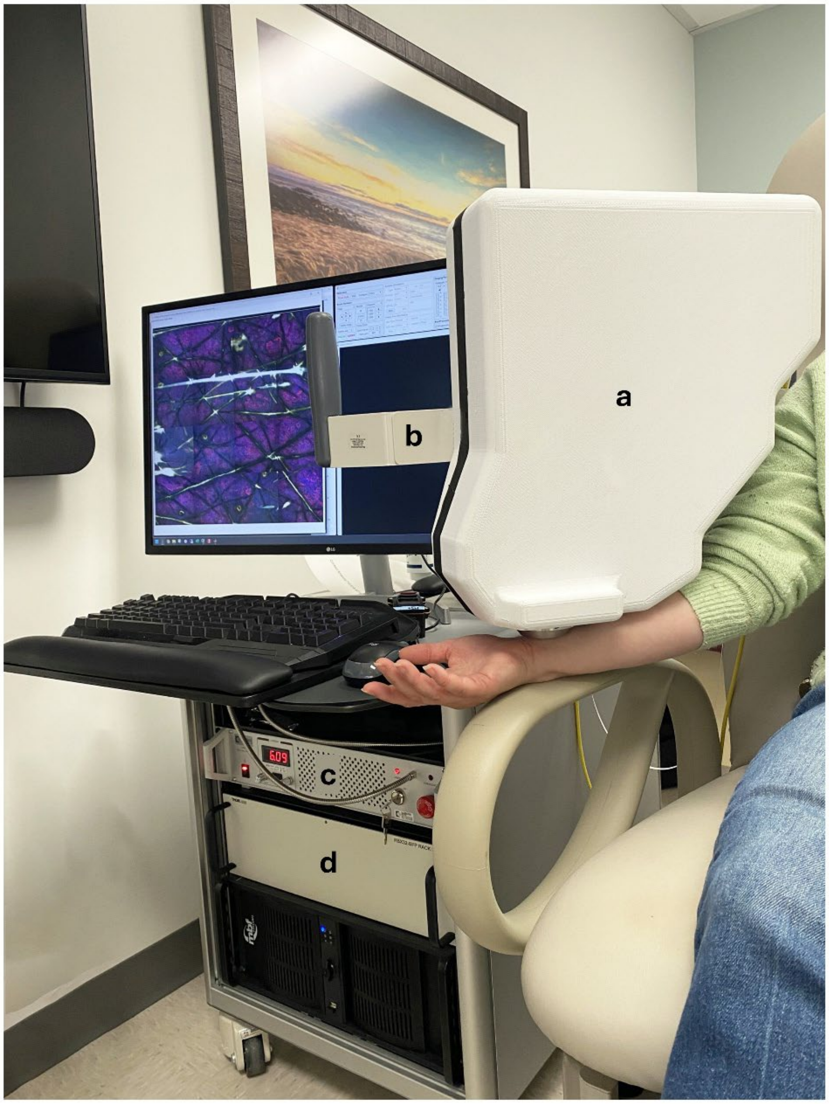

Supplemental Figure 1: Time resolved multiphoton imaging using the fast, large-area multiphoton exoscope (FLAME). The optical components of the FLAME system are housed in the microscope head (**a,**white polygonal box). The exoscope head can be manouvered in different angles using the handle (**b**). The head (**a**) is connected to the cart by an articulated arm allowing the system to be extended from the cart and positioned in different angles to the cart (not shown). The laser (**c**) from the calamar laser is delivered to the exoscopes head using an optical fiber. The electronic components are housed in the box (**d**).

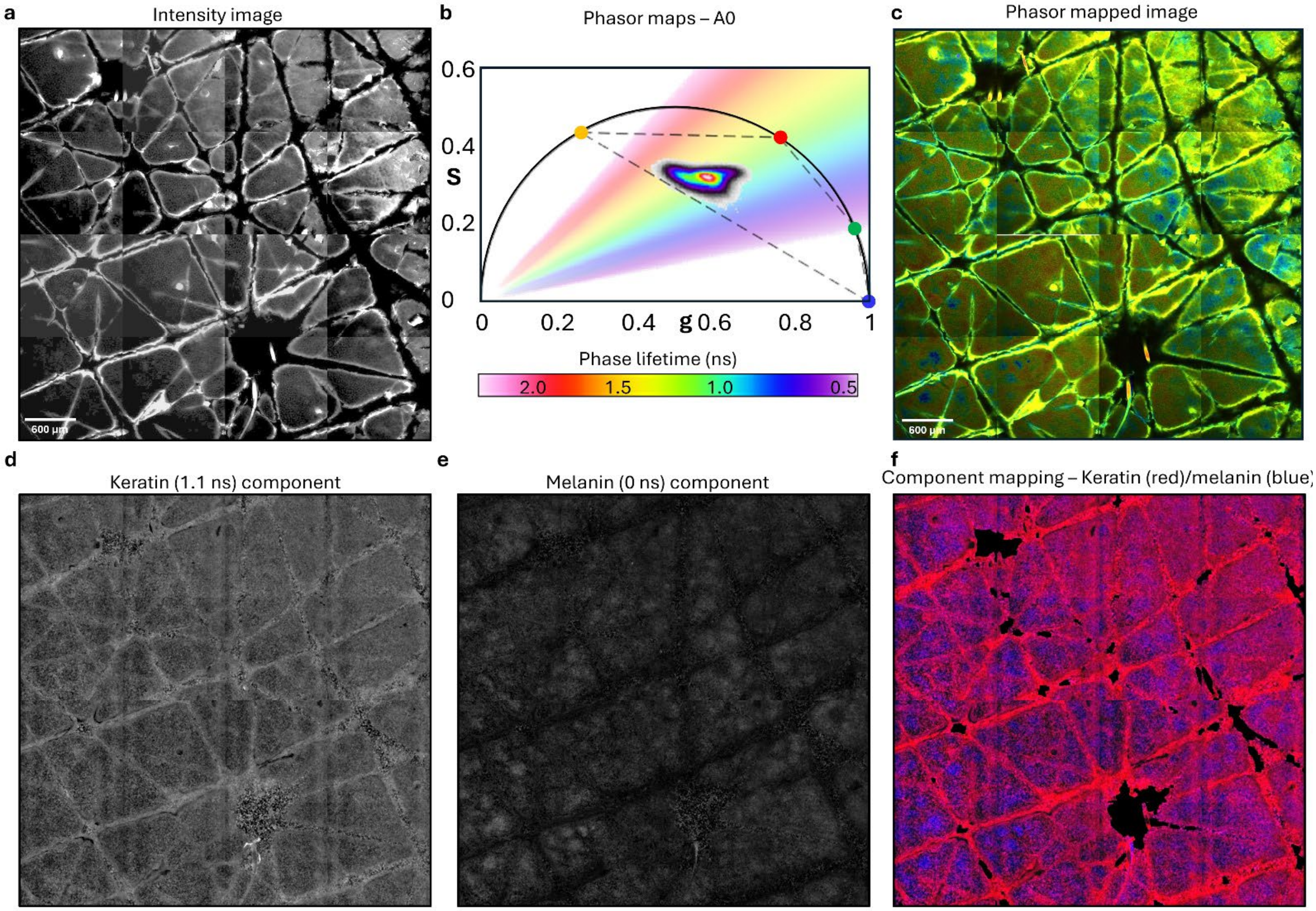

Supplemental Figure 2: Mapping Keratin and Melanin Distribution Over mm-scale Areas. a. Intensity image acquired *in vivo* at the basal layer depth using FLIM mode, over a 2.4 mm x 2.4 mm of skin in a volunteer with skin type III. **b.** Corresponding fluorescence lifetime phasor plot, with gradient color distribution representing tau-phase values. **c.** Phasor-colored image showing the distribution of keratin (yellow-green) and pigmented areas (blue), which correspond to longer and shorter fluorescence lifetimes, respectively. **d-e.** Fractional intensity maps for keratin (d) and melanin (e) distribution, alongside a combined color-coded map (f), that highlights significant keratin content in skin folds (d) and of melanin accumulation in the basal layer keratinocytes (e). This is further emphasized in the combined image, where keratin is represented in red and melanin in blue (f). Scale bar is 600 µm.

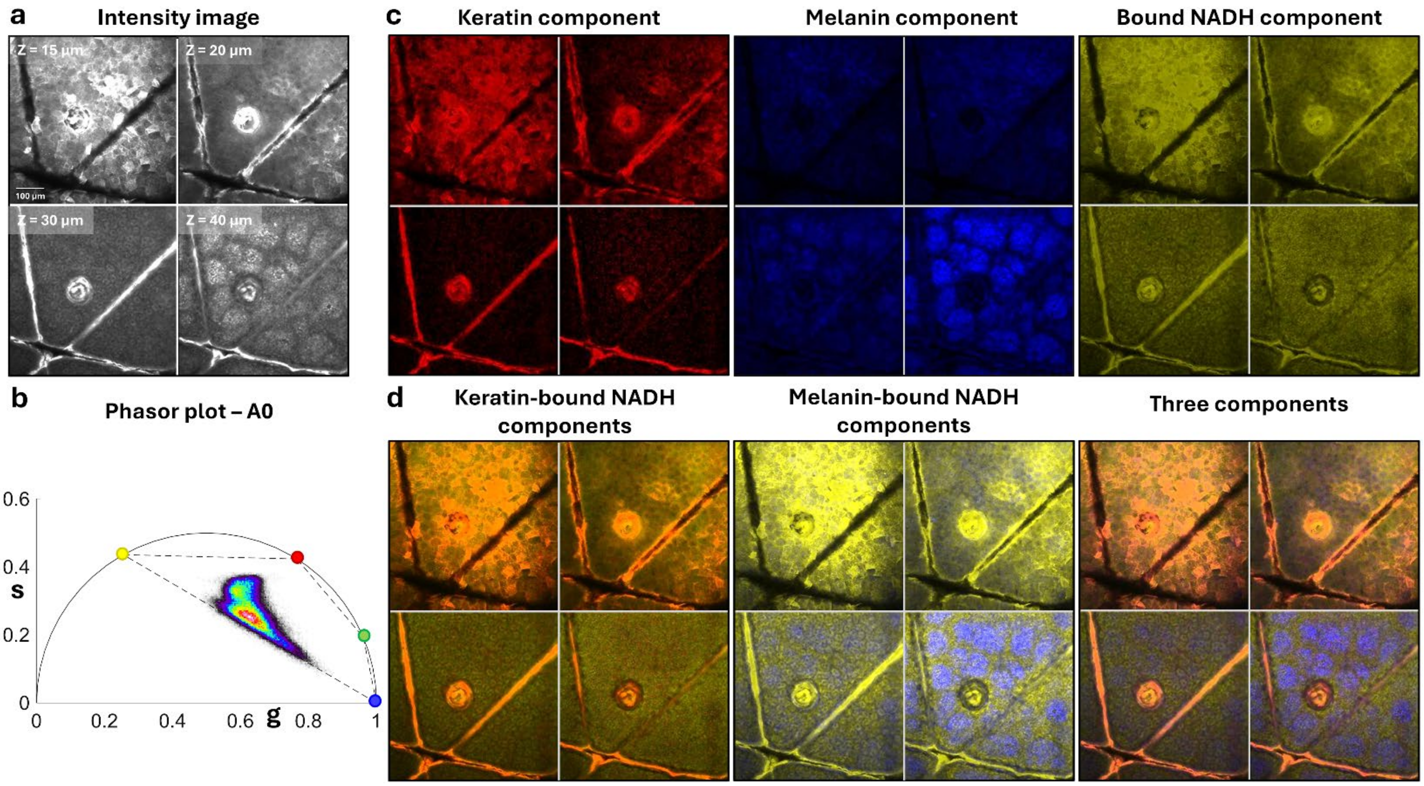

Supplemental Figure 3: Maps of Keratin, Melanin and Protein-Bound NADH from Skin Type V. a, Depth-resolved intensity images acquired *in vivo* from a subject with skin type V. **b,** Corresponding phasor plots. Colored circles indicate the component positions: protein-bound NADH (yellow), keratin (red), free NADH (green), and melanin (blue), arranged in order of decreasing phase angles. **c,** Individual component maps for keratin (red), melanin (blue), and protein-bound NADH (yellow), highlighting the absence of protein-bound NADH in the nucleus compared to the cytoplasm of keratinocytes. **d,** Two-component color maps for keratin and protein-bound NADH (left), melanin and protein-bound NADH (center), as well as three-component map for keratin, melanin, and protein-bound NADH (right). These maps illustrate the variations of different components as depth in the epidermis increases. Scale bar is 100 µm.

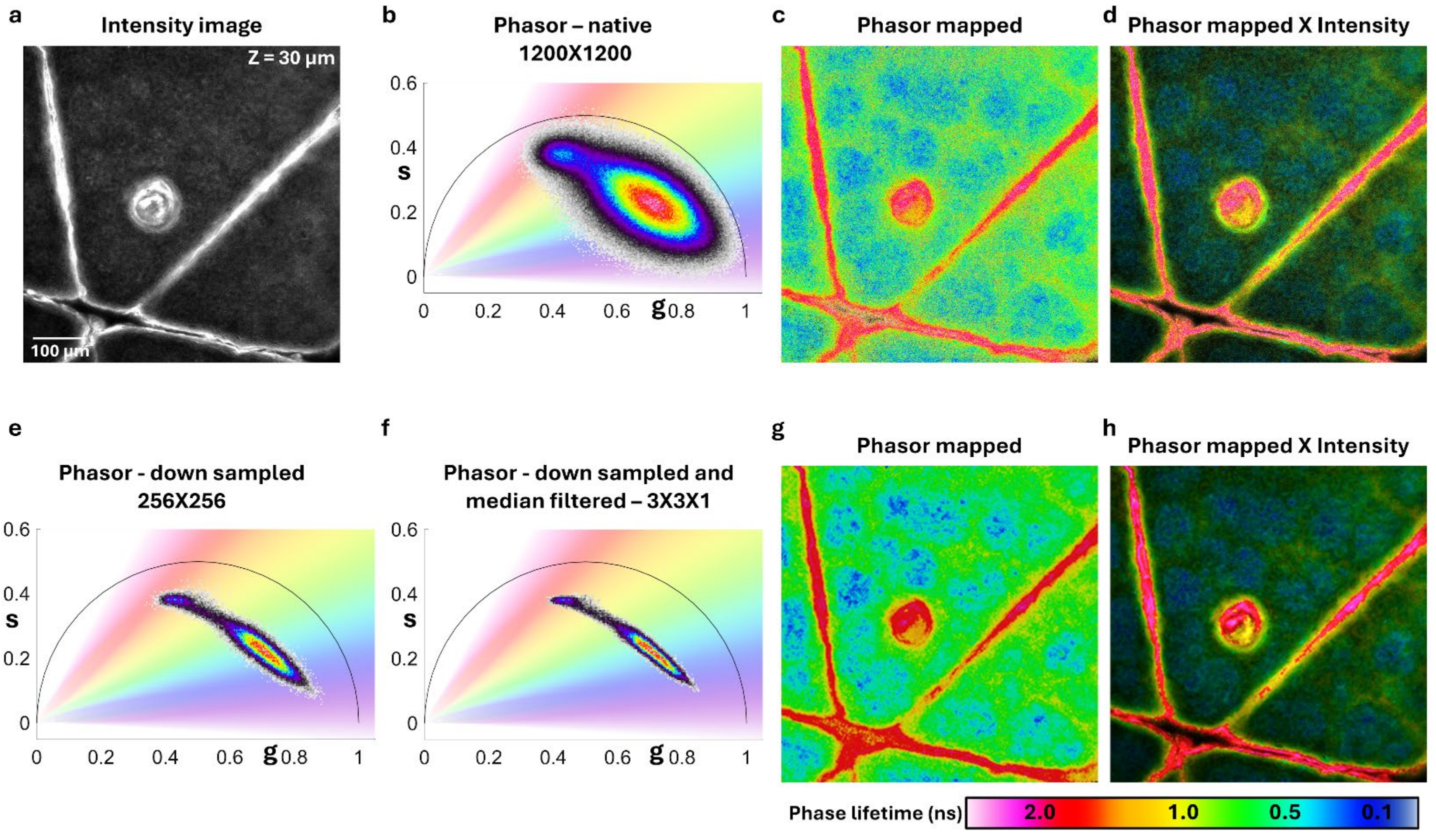

Supplemental Figure 4. Pipeline for FLIM data visualization and analysis. a, Intensity image displayed in GSLab at full resolution (1024 × 1024 pixels). **b,** Corresponding phasor plot without downsampling or filtering. **c,** Phasor-mapped image showing color-coded distribution in phasor space. **d,** Phasor-mapped image overlaid with the original intensity image. **e,** Reduced phasor spread following downsampling to 256 × 256 pixels. **f,** Application of a 3 × 3 median filter in phasor space to further reduce noise. **g,** Phasor-mapped image after downsampling and filtering. **h,** Final phasor-mapped image overlaid with the intensity image, corresponding to the original image in (a). Scale bar: 100 µm.

Supplemental table

Species	Lifetime (ns)	G	S
**Free NADH**	0.4 ns	0.96	0.20
**Protein-bound NADH**	3.4 ns	0.25	0.44
**Keratin**	1.1 ns	0.77	0.42
**Melanin**	0.0 ns	1.00	0.00

Supplemental Table 1. G,S coordinates from the measured and published data. The G.S coordinates, calculated from measurement *in vitro* (free and protein bound NADH) and *in vivo (*Keratin and Melanin), were used to calculate the component distribution.
