# Supplementary material for: Label-free human skin imaging with enhanced molecular contrast via time-resolved fluorescence and advanced phasor analysis
Source: Commun Biol. 2025 Dec 30;9:149. doi: 10.1038/s42003-025-09427-4 (PMC12868790; doi:10.1038/s42003-025-09427-4)
Supplement: Supplementary file 3 — Description of Additional Supplementary Files [file 42003_2025_9427_MOESM3_ESM.pdf]

Description of Additional Supplementary File

File name: Supplementary data 1
Description: Supplementary data that was used to create the violin plots in Figures 4 and 6

File name: Supplementary data 2
Description: Supplementary data that was used to create the violin plots in Figure 7
